# HRG inhibits liver cancer lung metastasis by suppressing neutrophil extracellular trap formation

**DOI:** 10.1002/ctm2.1283

**Published:** 2023-05-30

**Authors:** Yanze Yin, Huijuan Dai, Xicheng Sun, Zhifeng Xi, Jiang Zhang, Yixiao Pan, Yongxin Huang, Xueyun Ma, Qiang Xia, Kang He

**Affiliations:** ^1^ Department of Liver Surgery Renji Hospital School of Medicine Shanghai Jiao Tong University Shanghai China; ^2^ Shanghai Engineering Research Center of Transplantation and Immunology Shanghai China; ^3^ Shanghai Institute of Transplantation Shanghai China; ^4^ Department of Breast Surgery Renji Hospital School of Medicine Shanghai Jiao Tong University Shanghai China; ^5^ Shanghai Key Laboratory of Regulatory Biology Institute of Biomedical Sciences and School of Life Sciences East China Normal University Shanghai China

**Keywords:** liver cancer, lung metastasis, neutrophil, neutrophil extracellular trap

## Abstract

**Background:**

Distant metastasis is a sign of poor prognosis for cancer patients. Extrahepatic liver cancer metastases commonly spread to the lung. Remodelling of the metastatic microenvironment is essential for tumour metastasis. Neutrophil‐associated metastatic microenvironment contributes to the early metastatic colonisation of cancer cells in the lung.

**Method:**

The lung metastasis models were constructed via treated cancer cells by tail vein injection into mice. And samples of lung were harvested at the indicated time to analyze tumor growth and immune cells in the microenvironment. Tumors and lung metastasis specimens were obtained via surgical operations for research purposes. Neutrophils were obtained from peripheral blood of patients with liver cancer or healthy donors (HD).

**Results:**

Hepatocellular carcinoma cells reduce the secretion of histidine‐rich glycoprotein (HRG), regulate the recruitment and activation of neutrophils in the metastatic microenvironment and promote the production of neutrophil extracellular traps (NETs), thereby promoting liver cancer lung metastasis. HRG binds to FCγR1 on the neutrophil membrane while inhibiting PI3K and NF‐κB activation, thereby reducing IL‐8 secretion to reduce neutrophil recruitment. Meanwhile, HRG inhibited IL8‐MAPK and NF‐κB pathway activation and ROS production, resulting in reduced NETs formation.

**Conclusions:**

Our study reveals that liver cancer regulates neutrophil recruitment and NETs formation in the metastatic microenvironment by reducing HRG secretion, thereby promoting tumour lung metastasis. The results of this study will contribute to the development of possible strategies for treating metastases.

## Graphical abstract

- Decreased secretion of HRG by liver cancer.
- HRG suppresses NETs production from neutrophils.
- NETs promote liver cancer lung metastasis.

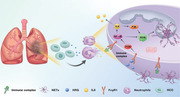

## 1 INTRODUCTION

According to the latest cancer statistics,^1^ hepatocellular carcinoma (HCC)‐related annual mortality rates have increased significantly over the past 20 years, and lung metastases are among the leading causes of hepatocellular liver cancer‐related mortality. In addition to intrinsic characteristics, tumour metastasis is linked to the tumour microenvironment, which contains a number of different types of cellular and noncellular components.^2^ Metastasis of primary tumours requires a favourable microenvironment, called the pre‐metastatic niche, and the secreted proteins of tumour cells are important mediators in reshaping the tumour microenvironment and maintaining the metastatic niche.^3^ However, little is known about the mechanism by which secreted proteins produced by tumour cells reshape the metastatic microenvironment.

Neutrophils are the first responders to inflammation and infection,^4^ but a growing number of studies suggest that neutrophils are also important players in the development of cancer.^5^, ^6^ In recent studies, neutrophils and neutrophil‐derived molecules have been shown to play a significant role in the formation of the pre‐metastatic niche and the entire transfer process. Neutrophils produce a special web‐like structure named neutrophil extracellular traps (NETs).^7^ NETs can capture circulating tumour cells and enable their adhesion to metastasize to distant sites.^8^ In addition, NETs can promote cancer metastasis by binding to CCDC25, a receptor on the surface of cancer cells.^9^ Recent studies have shown that NETs production by neutrophils can promote liver cancer metastasis by triggering a tumorigenic inflammatory response.^10^ However, how tumour cells affect NETs production by neutrophils in the metastatic ecotone remains to be investigated.

Histidine‐rich glycoprotein (HRG) is a secretory glycoprotein, secreted primarily by hepatocytes, that binds to a variety of ligands including heme, heparin, heparan sulphate, platelet‐reactive protein, plasminogen and divalent metal ions.^11^ HRG is involved in regulating some physiological processes such as the immune complex and pathogen clearance, cell chemotaxis, cell adhesion, angiogenesis, coagulation and fibrinolysis. Previous studies have found that HRG plays an important role in liver disease by regulating macrophage polarisation,^12^, ^13^, ^14^ while the effect of HRG on tumour metastasis has not been studied.

In this study, we screened out metastasis‐associated secretory protein HRG through a public database and reported that HCC cells promote lung metastasis by reducing HRG secretion thus regulating infiltration of neutrophil and NETs formation in metastatic microenvironment.

## 2 RESULTS

### 2.1 HRG is associated with liver cancer lung metastasis

**FIGURE 1:**
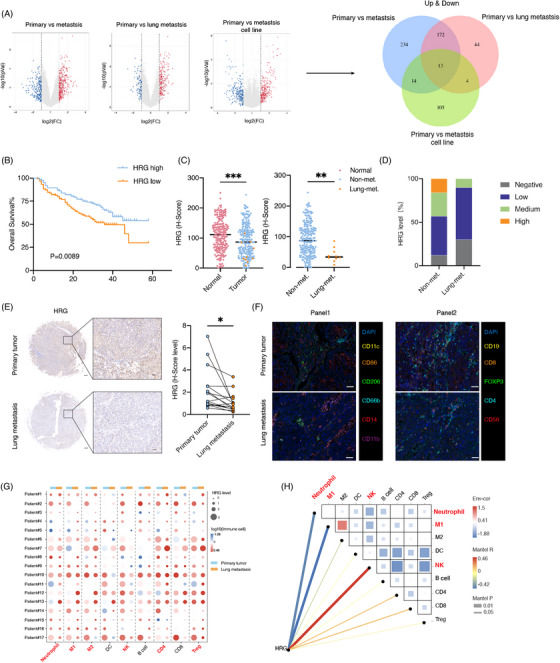
HRG secretion is associated with liver cancer lung metastasis immune microenvironment. (A) Volcano plots of differential gene expression in 10 primary liver cancer, 19 extrahepatic metastasis (13 lung metastasis, three adrenal gland metastasis and three lymph node metastasis), 13 lung metastasis, three primary cell lines and two metastasis cell lines. Blue and red dots represent down‐regulated and up‐regulated secreted protein genes, respectively. Veen diagram of differential secretory protein genes (Both up and down) in primary liver cancer, extrahepatic metastasis, lung metastasis, primary cell lines and metastasis cell lines. (B–D) HRG expression analysis of a liver cancer cohort from Shanghai Renji Hospital (*n* = 234). (B) Overall survival and (C) comparisons of HRG expression in normal (*n* = 234) with tumour (*n* = 234) and non‐metastasis (*n* = 224) with lung metastasis (*n* = 10). (D) HRG expression level in non‐metastasis (*n* = 224) with lung metastasis (*n* = 10). (E) Comparisons of HRG expression in patients (*n* = 17) with paired primary tumours and lung metastases. Scale bars, 200 and 50 μm. (F–H) Analyses of HRG expression and immune infiltration in paired primary tumours and lung metastases form the same patients (*n* = 17) by multi‐immunohistochemistry (mIHC) staining of metastasis‐associated myeloid and lymphatic immune cells. (F) Representative mIHC staining images of metastasis‐associated myeloid (Panel 1) and lymphatic immune cells (Panel 2). Scale bars, 50 μm. (g) Dot plots displaying HRG expression level and altered immune cells in paired primary tumours and lung metastases. (h) Correlation analyses between HRG expression and metastasis‐associated immune cells. *p < .05, **p < .01, ***p < .001, by log rank test (B) and two‐tailed unpaired (C) or paired (E) *t*‐test.

To find genes associated with lung metastasis of liver cancer, we used Gene Expression Omnibus Dataset (GSE40367) for bioinformatics analysis. Differential gene analysis of primary HCC and extrahepatic metastases and primary HCC and lung metastases were performed, respectively. In addition, we downloaded transcriptome data for HCC cell lines from the Cancer Cell Line Encyclopedia (https://sites.broadinstitute.org/ccle) for differential gene analysis of metastases cell lines (SK‐HEP‐1, JHH2) and primary cell lines (PLC/PRF/5, JHH1, HuH7) (Figure 1A). We focused particularly on the secreted proteins in these genes and identified a total of 13 secretory protein genes, namely CYBA, HRG, TRABD2A, THBS1, BACE2, TOX2, KNG1, SERPINE1, CA9, ADAMTS10, GAL3ST1, MME and ROR1, by intervening in the secretory protein difference genes of tissues and cell lines. HRG is the only liver‐specifically secreted protein (Figure S1a), and HRG was down‐regulated in metastasis compared to primary cell lines. In addition, we analysed the distribution of HRG in HCC and CCA using single‐cell RNA‐seq database. And we found that mainly hepatocytes and tumour cells expressed HRG (Figures S2a, b, c and d).

To further validate the clinical relevance and HRG expression, we analysed  a cohort of liver cancer for HRG immunohistochemical staining and found that lower HRG expression in primary tumours was associated with shortened overall survival, metastasis‐free survival and lung metastasis‐free survival in patients (Figures 1B and S1b), and lower HRG expression in primary tumours was associated with extrahepatic metastases and lung metastases (Figure S1c). The HRG protein level of primary liver cancer tissue is significantly lower than that of normal liver tissue, and the HRG protein level of liver cancer tissue in patients with lung metastases is significantly lower than in patients without lung metastases (Figures 1C and D). In addition, analysis of paired primary tumours and lung metastases from the same liver cancer patients with lung metastases (*n* = 17) further confirmed a significant reduction in HRG in lung metastases (Figure 1E). These results suggest that HRG is inversely associated with lung metastasis in liver cancer.

To study the effects of HRG on the metastatic immune microenvironment, we conducted a correlation analysis between HRG and immune cells through the TCGA database and found that the low HRG expression group had higher TIMER immune scores of neutrophil, myeloid DC, macrophage, CD4+T and B cell compared with the high HRG expression group (Figure S3a), and HRG was inversely correlated with neutrophil, myeloid DC, macrophage, CD4+T and B cells (Figures S3b and c). In addition, we examined immune cells associated with metastasis in primary and metastases tissues from the same patient through the fluorescent multiplex immunohistochemistry assay (Figure 1F), found that the number of neutrophils, M1, M2, CD4+ T and Treg cells increased and the number of NK cells decreased in lung metastases (Figures 1G and S3d). Additionally, we analysed the relationship of HRG and immune cells and found that HRG was negatively correlated with neutrophil and M1 cells, while positively correlated with NK cells (Figure 1H). These results suggest that HRG is associated with neutrophil, macrophage and NK cells in the metastatic microenvironment.

### 2.2 HRG reduced liver cancer lung metastasis in vivo

**FIGURE 2:**
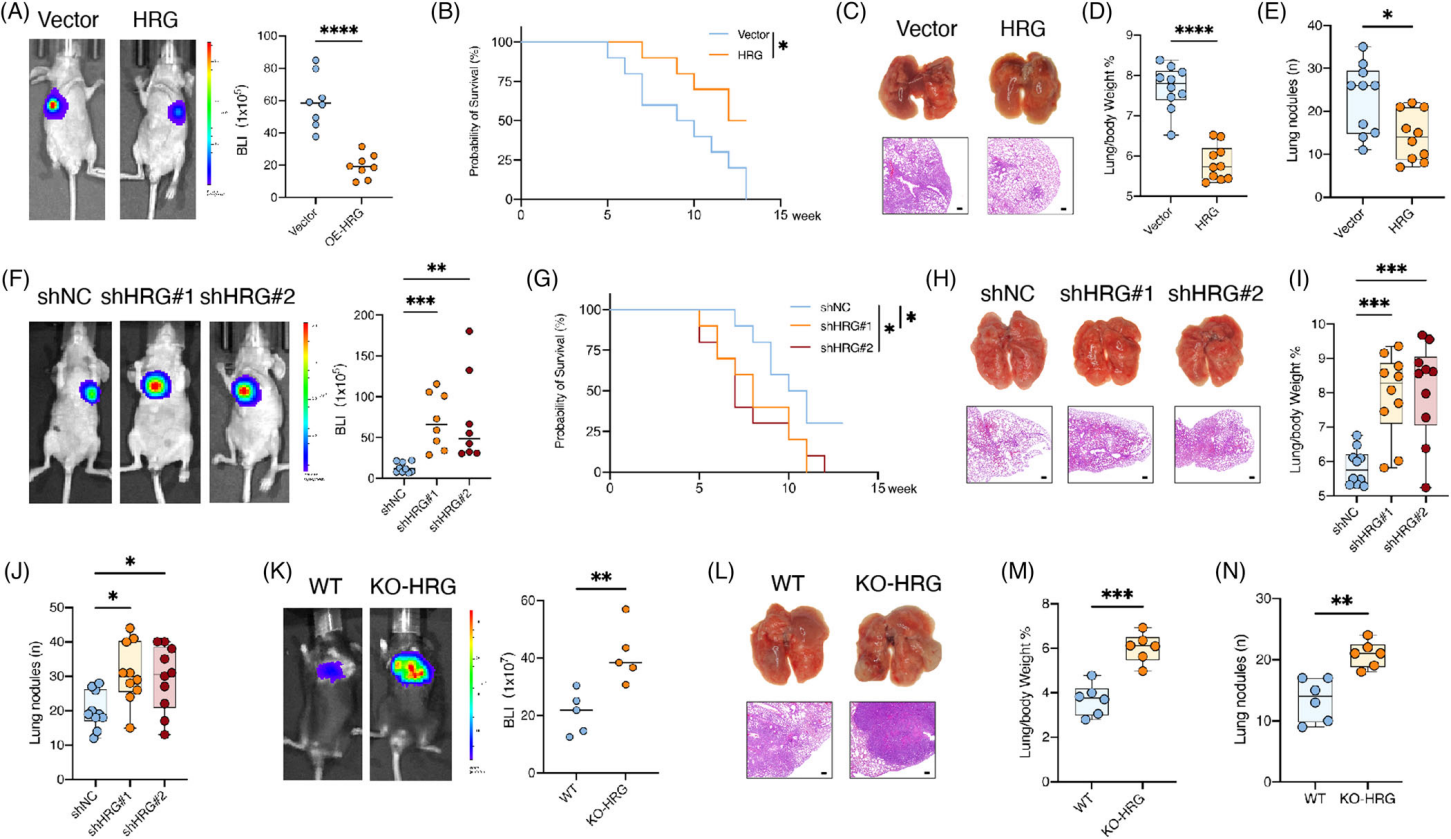
HRG reduced liver cancer lung metastasis. (A–E) Intravenous injection of SK‐hep1 with HRG overexpression in athymic mice for lung metastasis analysis. Samples of lung were harvested at 5 weeks after injection. Shown are (A) bioluminescent imaging (BLI) quantification and representative images (*n* = 8), (B) animal survival curve (*n* = 10), (C) representative macro and micro images of lung nodules (*n* = 10), and boxplots of (D) lung/body weight and (E) pulmonary surface nodules (*n* = 10). Scale bars, 50 μm. (F–j) Intravenous injection of HCC‐LM3 with HRG knockdown in athymic mice for lung metastasis analysis. Samples of lung were harvested at 5 weeks after injection. Shown are (F) BLI quantification and representative images (*n* = 8–9), (G) animal survival curve (*n* = 10), (H) representative macro and micro images of lung nodules (*n* = 10), and boxplots of lung/body weight (I) and pulmonary surface nodules (J) (*n* = 10). Scale bars, 50 μm. (K–N) Intravenous injection of Hepa 1−6 in C57BL/6 mice for lung metastasis analysis. Samples of lung were harvested at 5 weeks after injection. Shown are (K) BLI quantification and representative images (*n* = 5), (L) representative macro and micro images of lung nodules (*n* = 6), and (M) boxplots of lung/body weight and (*n*) pulmonary surface nodules (*n* = 6). Scale bars, 50 μm. *p < .05, **p < .01, ***p < .001, by log rank test (B and G) and two‐tailed unpaired *t*‐test (others).

We examined the expression of HRG in HCC cell lines (Figure S4a) and found that HRG expression was lower in SKHEP1 and higher in LM3. HRG overexpression in SKHEP1 (Figure S4b), reduced lung metastases of cancer cells (Figures 2A and c–E) and prolonged the survival of mice (Figure 2B). Conversely, HRG knockdown by short hairpin RNA in LM3 (Figure S4c), enhanced lung metastases of cancer cells (Figures 2F and H–J) and shortened the survival of mice (Figure 2G). HRG in conditioned medium (CM) of HCC cells and normal liver cells was detected by ELISA (Figure S4d).

To investigate the role of HRG in mice with intact immune activeness, WT and KO‐HRG mice were tail vein injected with mouse HCC cell line Hepa 1−6, and we found that KO‐HRG mice showed significantly higher intrapulmonary metastatic tumour loads than WT mice (Figures 2K–N).

We studied the effect of HRG on cancer cells and found that HRG overexpression or knockdown did not affect the proliferation, invasion and migration ability of cancer cells in vitro (Figures S5a–c), which suggests that the regulatory role of HRG on lung metastasis of liver cancer depends on the metastatic microenvironment.

### 2.3 HRG decreases neutrophil in early lung metastatic microenvironment

**FIGURE 3:**
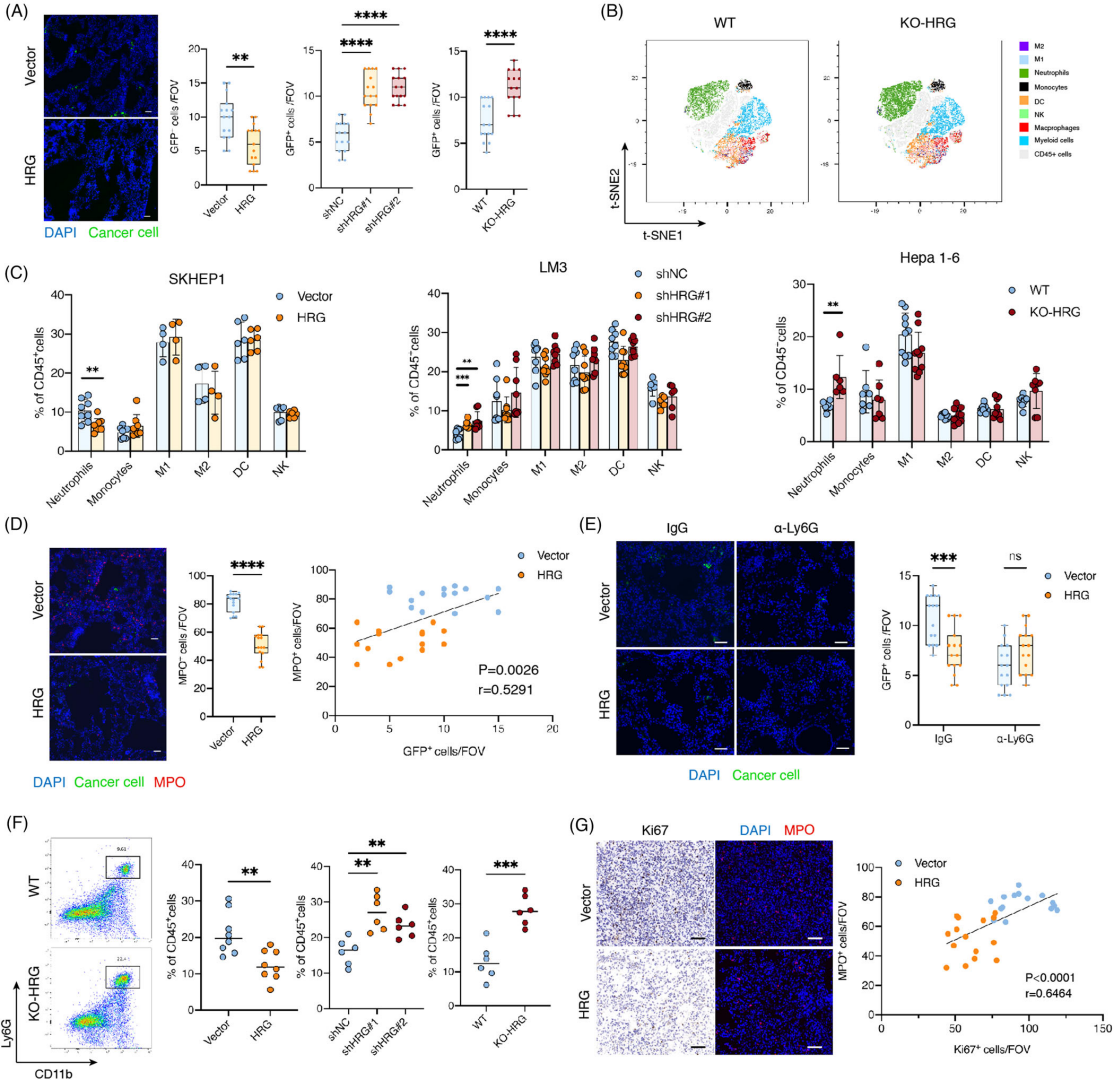
HRG decreases neutrophil in lung metastasis niches. (A) Immunofluorescent (IF) staining analyses of GFP+ cancer cells at the early stage after intravenous injection. Shown are representative images of GFP+ SK‐hep1 and boxplots showing GFP+ cancer cells in lung at 72 h after intravenous injection (*n* = 15 random microscopic fields (RMFs) from three mice of each group). Scale bars, 50 μm. (B–D) Flow cytometry analyses of alternations of immune cells in early lung metastases (72 h after intravenous injection). (B) Representative t‐SNE maps and (C) statistical graph showing percentage of immune cells. (*n* = 5–10). (D) IF analyses of MPO+ neutrophils and correlation of infiltrated MPO+ neutrophils with GFP+ cancer cells at 72 h after intravenous injection (*n* = 15 RMFs from three mice per group). Scale bars, 50 μm. (E) Quantification of GFP+ cancer cells in lung of mice treated with IgG or the neutrophil clearance antibody at 72 h after intravenous injection of SK‐hep1 (*n* = 15 RMFs from three mice per group). Scale bars, 50 μm. (F) Flow cytometry analyses of CD11b+Ly6G+ neutrophils in lung metastases at 5 w after intravenous injection (*n* = 6). (G) Correlation of MPO+ neutrophils and Ki67+ cancer cells in lung metastases at 5 w after intravenous injection (*n* = 15 RMFs from three mice per group). Scale bars, 50 μm. *p < .05, **p < .01, ***p < .001, by Pearson correlation analysis (D and G) and two‐tailed unpaired *t*‐test (others).

We explored which stages of HRG affected the metastatic colonisation of cancer cells. Immunofluorescence staining revealed a significant reduction in the colonisation of HRG‐overexpressed cancer cells in the lungs 3 days after tail vein injection. Consistently, HRG knockdown cancer cells and cancer cells in KO‐HRG mice seeding increased (Figure 3A), indicating that HRG plays a critical role in the early metastatic stage. We further explored the effects of HRG on the pulmonary immune microenvironment in the early metastatic phase. By flow cytometry assays, we found that HRG overexpression cancer cells inhibited CD11b+Ly6G+ neutrophils, while HRG knockdown cancer cells and KO‐HRG mice enhanced the percentage of CD11b+Ly6G+ neutrophils rather than other immune cells (Figures 3B and C). Immunofluorescence staining of lung tissue further confirmed that neutrophils around HRG overexpression cancer cells increased, and neutrophils around HRG knockdown cancer cells and cancer cells in KO‐HRG mice decreased. In addition, we observed a positive correlation between early metastatic cancer cells and neutrophils (Figures 3D and S6a,b). These results suggest that HRG could reduce the number of neutrophils in the lung metastatic microenvironment. In addition, we depleted neutrophils in mice with anti‐Ly6G antibody (Figure S6c) and found that the lung metastatic regulation effect of HRG on cancer cells was eliminated (Figure 3E). We further explored the number of neutrophils after tumorigenesis in the lungs by flow cytometry and found that the number of neutrophils in HRG overexpressed tumours decreased and the number of neutrophils in HRG knockdown tumours and KO‐HRG mice increased (Figure 3F). Immunofluorescence staining experiments for lung metastases also confirmed the inhibitory effect of HRG on neutrophils in lung metastases, and we observed a positive correlation between the number of Ki67+ cancer cells and neutrophils (Figures 3G and S6d and e). These results suggest that the regulatory role of HRG in lung metastasis of cancer cells is associated with neutrophils.

### 2.4 HRG suppresses neutrophil recruitment and activity through FCγR1 of neutrophils

**FIGURE 4:**
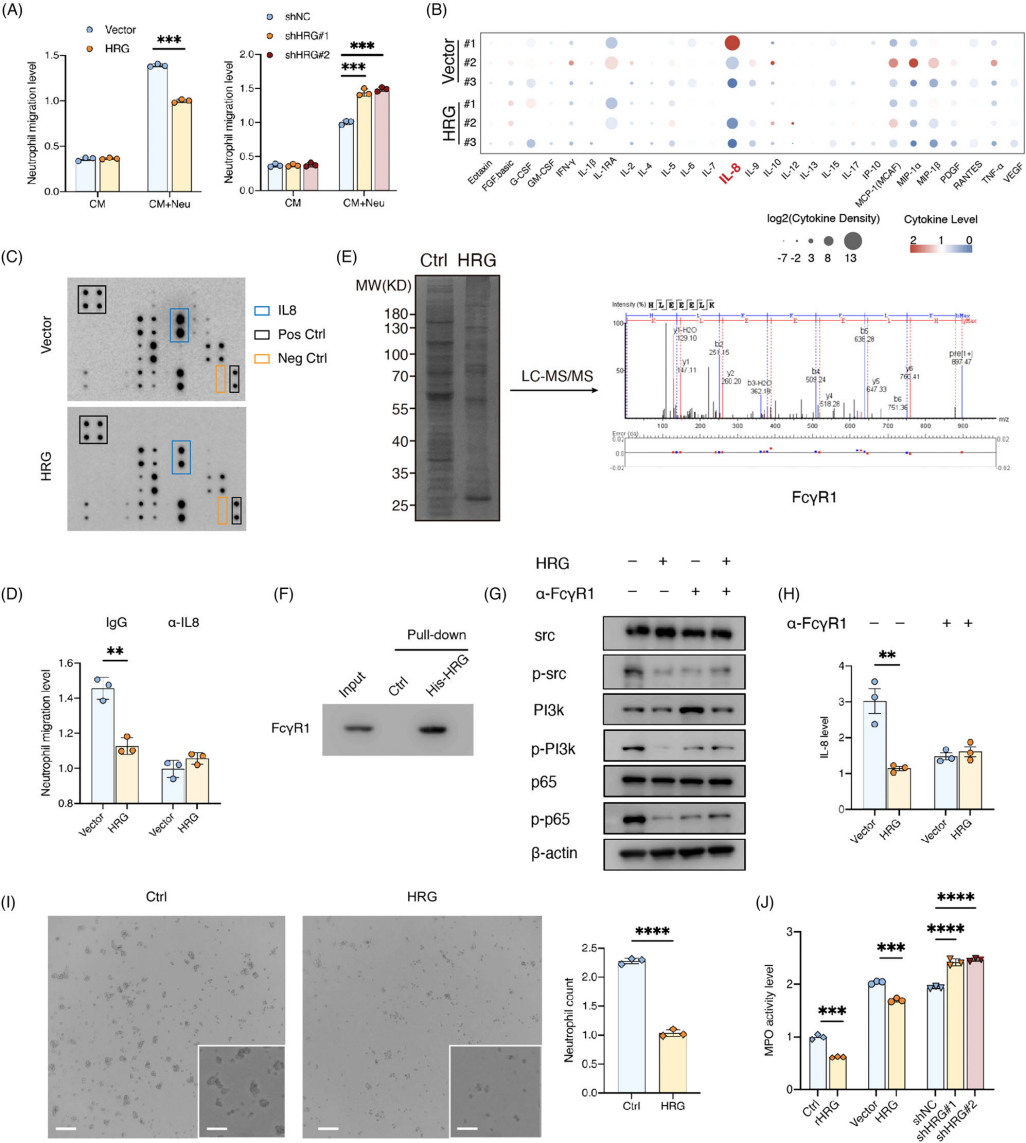
HRG suppresses neutrophil recruitment and activity through FCγR1 of neutrophil. (A) Migration of human neutrophils recruited by conditioned medium (CM) of SK‐hep1 (HRG overexpression) or LM3 (HRG knockdown), or by medium from neutrophils (Neu) pre‐treated with CM for 8 h (*n* = 3). (B) Cytokine analyses in media of neutrophils from liver cancer patients pre‐treated with CM of SK‐hep1 (HRG overexpression) for 8 h (*n* = 3). (C) Cytokine array analyses in media of neutrophils from HDs pre‐treated with CM for 8 h from three donors (*n* = 3). (D) Migration of human neutrophils recruited by CM+Neu medium of SK‐hep1 (HRG overexpression) treated with IgG or the IL‐8 clearance antibody for 8 h (*n* = 3). (E) His‐pulldown assay of human neutrophils membrane protein treated with His‐HRG for 8 h and liquid chromatography with mass spectrometry/mass spectrometry (LC–MS/MS) analysis. (F) His‐pulldown assay consolidating HRG could combine FCγR1 in the human neutrophil membrane. (g) Src phosphorylation, PI3K phosphorylation and p65 phosphorylation in human neutrophils pre‐treated with CM or FCγR1 blocking antibody. (H) ELISA assay of IL‐8 from human neutrophils treated with CM or IL‐8 blocking antibody (*n* = 3). (I) Human neutrophils pre‐treated with CM for 8 h (*n* = 3). Scale bars, 50 μm. (J) MPO activity of human neutrophils pre‐treated with rHRG and CM (*n* = 3). *p < .05, **p < .01, ***p < .001, by two‐tailed unpaired *t*‐test.

We continued to explore how HRG affects neutrophil recruitment. HRG overexpression or knockdown of cancer cells did not alter the attraction effect of CM on neutrophils. However, when human peripheral blood neutrophils were pre‐treated with a cancer cell CM containing HRG, the number of neutrophils attracted by the medium of neutrophils was significantly reduced, and by contrast, the neutrophil medium pre‐treated with CM of HRG knockdown attracted more neutrophil migration (Figure 4A), indicating that HRG may act on neutrophils and regulate the secretion of related chemokines to impact the recruitment of neutrophils. In order to determine the cytokines released by the HRG effect on neutrophils, we treated three cases of neutrophils from the peripheral blood of patients with HCC with cancer cell CM, and then detected cytokines in neutrophil supernatants, and found that the IL‐8 content of the three patients was reduced (Figure 4B). In addition, we analysed the effect of overexpressed HRG cancer cell CM on neutrophil cytokines in healthy donors (HDs) with a cytokine antibody array and found that HRG overexpressed cancer cell CM reduced the IL‐8 released by neutrophils (Figure 4C). Previous studies^15^, ^16^ have shown that IL‐8 has the effect of chemotactic neutrophils, and we have also found that the use of anti‐IL‐8 antibodies can eliminate the inhibitory recruitment effect of HRG on neutrophils (Figure 4D). These data suggest that HRG can inhibit neutrophil chemotaxis by reducing the release of IL8 from neutrophils.

To further explore the molecular mechanisms of HRG acting on neutrophils, we extracted membrane proteins from neutrophils derived from human peripheral blood and pull‐down His‐HRG after co‐culture with HRG. Liquid chromatography with mass spectrometry/mass spectrometry analysis found that the membrane protein FCγR1 on the membrane of neutrophils may be a receptor for HRG (Figure 4E). Previous studies^17^, ^18^ have supported this result. Further pull‐down experiments confirmed that HRG can bind to FCγR1 (Figure 4F). Studies^19^, ^20^ have found that FCγR1 maintains the activated state of neutrophils by PI3K after binding to the immune complex. In addition, IL‐8 is also a downstream target for the known PI3K‐NF‐κB signalling pathway,^21^, ^22^, ^23^ and we found that HRG inhibits the conduction of the PI3K and NF‐κB signalling pathways and reduces the production of downstream IL‐8. We found that the use of anti‐FCγR1 antibody eliminated the effects of HRG on the PI3K and NF‐κB signalling pathways and IL‐8 (Figures 4G and H), further demonstrating that HRG inhibits PI3K and the NF‐κB signalling pathway by binding to the FCγR1 receptor and thus affects neutrophil chemotaxis. In addition, because FCγR1 is critical for neutrophils to maintain viability, we explored the effect of HRG on neutrophil activity and found that rHRG significantly inhibited neutrophils and reduced neutrophil activity. Consistently, HRG overexpressed cancer cell CM reduced the MPO activity and the number of neutrophils and the MPO activity of neutrophils treated with HRG knockdown cancer cell CM was higher (Figures 4I and J). These data show that HRG secreted by cancer cells into the metastatic microenvironment can inhibit the PI3K‐NF‐κB signalling pathway of neutrophils through FCγR1, reducing the production of IL‐8 and thus reducing the recruitment of neutrophils. In addition, HRG further amplifies the regulatory effect by killing neutrophils.

### 2.5 HRG regulates liver cancer lung metastasis by inhibiting neutrophils to form NETs

**FIGURE 5:**
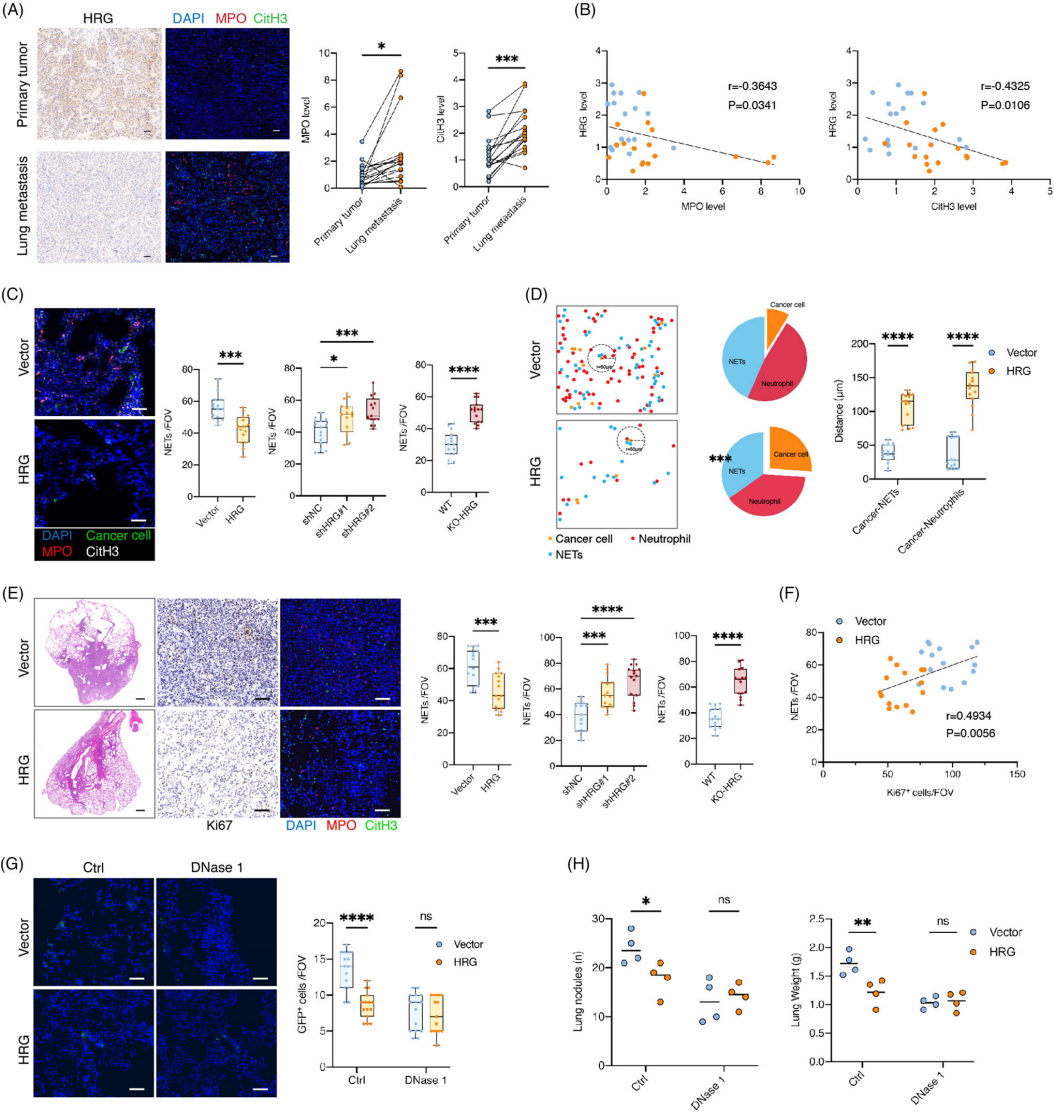
HRG regulates liver cancer lung metastasis by inhibiting neutrophils to form NETs . (A and B) IHC analysis of HRG level and IF analysis of MPO and CitH3 level in patients (*n* = 17) with paired primary tumours and lung metastases. (A) Comparisons of MPO and CitH3 level in primary and metastatic tumours from the same patient. (B) Correlation analysis of HRG expression with MPO and CitH3 level. Scale bars, 50 μm. (C) IF analyses of NETs around GFP+ cancer cell in the lung at 72 h after intravenous injection (*n* = 15 RMFs from three mice per group). Scale bars, 50 μm. (D) Spatial organisation patterns of cancer cells (orange), MPO+ neutrophils (red) and NETs (blue) in the tumour microenvironment. Pies show the percentage of MPO+ neutrophils and NETs next to cancer cells and mean spatial distance analyses of MPO+ neutrophils, NETs and cancer cells. (E and F) Lung metastasis at 5 weeks after injection of SK‐hep1 (HRG overexpression). (E) (Left) Representative images of lung metastasis, IHC and IF staining. (Right) Quantification of NETs formation (*n* = 15 RMFs from three mice per group). Scale bars, 50 μm. (F) Correlation analysis of NETs formation and proliferative cancer cells (Ki67+ cells). (G and H) Lung metastases burden of mice treated with DNase1 after injection of SK‐hep (HRG overexpression). (G) IF analyses of GFP+ cancer cells in the lungs at 72 h after injection of SK‐hep (*n* = 15 RMFs from three mice per group). and (H) lung metastases burden at 5 weeks after injection of SK‐hep (*n* = 4). Scale bars, 50 μm. *p < .05, **p < .01, ***p < .001, by Pearson correlation analysis (B and F) and two‐tailed paired (A) *t*‐test or unpaired *t*‐test (others).

We investigated how neutrophils in metastatic niches regulate the metastasis of cancer cells. Previous studies^24^, ^25^ have shown that neutrophils can be induced by cancer cells to form NETs that facilitate metastasis. We performed immunohistochemistry and immunofluorescence staining of tissue sections of paired primary tumours and lung metastases and found that the number of neutrophils in lung metastases and the levels of NETs produced were higher than those of primary tumours (Figures 5A and S7a), and we observed that HRG was inversely correlated with both neutrophils and NETs (Figure 5B). In addition, we analysed HRG, neutrophils and NETs in nine extrahepatic metastases (six bone metastases and three abdominal metastases) and found that HRG was inversely correlated with both neutrophils and NETs (Figures S7b and c). And we performed immunohistochemistry and immunofluorescence staining of tissue sections in another independent cohort of patients with liver cancer (*n* = 80) and found that HRG was inversely correlated with both neutrophils and NETs (Figures S7d and e).

To explore whether HRG regulates neutrophil NETosis, we explored the effect of HRG on lung NETs in the early metastasis stage and found that NETs around HRG overexpressed cancer cells decreased, while NETs around HRG knockdown cancer cells and cancer cells in KO‐HRG mice increased (Figure 5C). We also observed the spatial distribution of cancer cells with neutrophils or NETs and found that neutrophils and NETs around HRG overexpressed cancer cells decreased, and the distance between HRG overexpressed cancer cells and the nearest neighbour of neutrophils and NETs increased (Figure 5D). These results suggest that HRG can inhibit neutrophil NETosis during the early metastases phase of cancer cells. We further explored the NETs after lung tumorigenesis and found that NETs in lung metastases overexpressed with HRG decreased, while NETs in lung metastases with HRG knockdown and KO‐HRG mice increased (Figure 5E), while Ki67+ cancer cells were positively correlated with NETs (Figures 5f and S7f and g). Therefore, we injected mice with DNase 1 in advance to deplete the NETs and found that the effect of HRG on lung metastasis of cancer cells was eliminated in the early metastatic stage of cancer cells and after lung tumorigenesis (Figures 5G and H). These results suggest that NETs play a key role in HRG regulating lung metastasis of cancer cells.

### 2.6 HRG suppresses NETs formation by inhibiting NF‐κB and MAPK signalling pathways and declining ROS production

**FIGURE 6:**
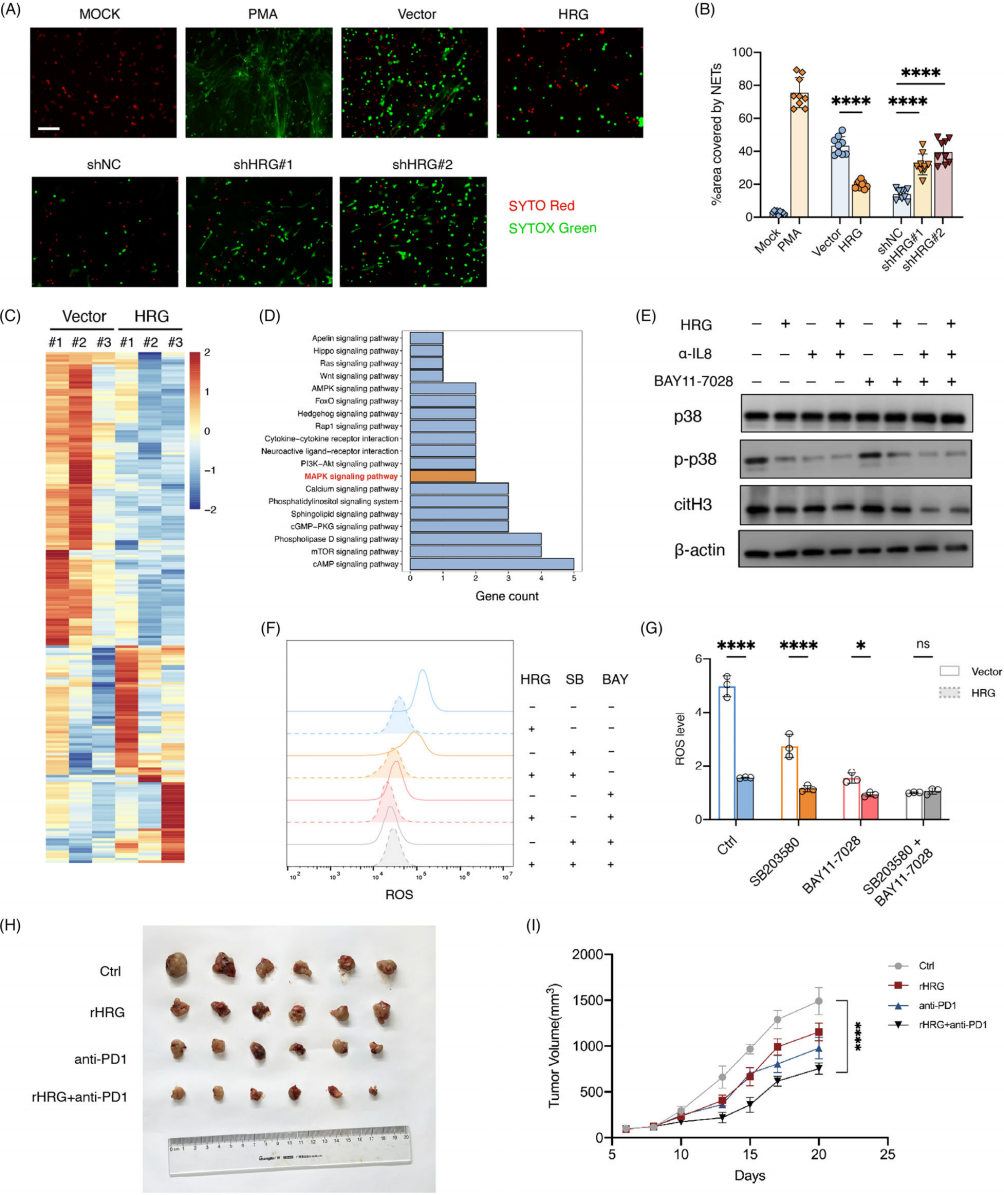
HRG suppresses NETs formation via inhibiting NF‐κB and MAPK signalling pathway and declining ROS production. (A and B) NET formation by Human neutrophils treated with PMA or CM from SK‐hep1 or LM3. (A) Representative images of NETs staining with cell‐permeable SYTO Red and cell‐impermeable SYTOX Green and (B) quantification of area covered by NETs (*n* = 9 RMFs from three biologically independent samples). Scale bars, 50 μm. (C and D) RNA‐sequence of human neutrophils treated with CM from SK‐hep1 (Vector and HRG overexpression) for 8 h. (C) Heatmap of differential expressed genes and (D) KEGG pathway analysis. (E) p38 phosphorylation and citH3 in neutrophils treated with CM from SK‐hep1, and IL8 blocking antibody or inhibitor of NF‐κB (BAY11‐7028). (f and g) ROS level of neutrophils treated with CM from SK‐hep1, and IL8 blocking antibody or inhibitor of NF‐κB (BAY11‐7028) or inhibitor of p38 (SB203580). (H and I) Hepa1‐6 cells were inoculated into C57BL/6 mice to construct subcutaneous graft tumours. Mice were distributed into four groups: anti‐PD‐1 monoclonal antibody combined with rHRG group, anti‐PD1 monoclonal antibody group, rHRG group and control group. Tumour samples were harvested after 3 weeks. (H) Tumour size and (I) growth curve. *p < .05, **p < .01, ***p < .001, by two‐tailed unpaired *t*‐test.

We used SYTO Red and SYTOX Green dyes staining to further confirm the effect of HRG on the formation of NETs (Figures 6A and B). We further explored the molecular mechanism by which HRG inhibits NETs formation of neutrophils. Reactive oxygen species (ROS) are known to play a key role in NETosis,^26^, ^27^ and we found that HRG inhibits ROS levels in neutrophils (Figures 6F and G). The MAPK pathway could be activated by IL‐8 to trigger ROS production,^24^, ^28^ and we found that HRG could regulate IL‐8 production of neutrophils. Consistently, we performed RNA sequencing and differentially expressed gene enrichment analysis on human neutrophils treated with CM of HRG overexpressed cancer cells, and the results showed that the MAPK signalling pathway of neutrophils treated with HRG overexpressed cancer cells CM was altered (Figures 6C and D). We found that HRG inhibited phosphorylation level of p38 in neutrophils, which could be eliminated by an anti‐IL‐8 antibody, but anti‐IL‐8 antibody did not completely eliminate the effects of HRG on citH3 (Figure 6E), which suggests that HRG does not regulate neutrophil NETs through the MAPK pathway only. The NF‐κB signalling pathway can also induce ROS production in neutrophils,^29^, ^30^ and we found HRG had a regulatory effect on the NF‐κB signalling pathway. To further explore the regulatory mechanism of HRG on neutrophils, we used MAPK inhibitors (SB203580) and NF‐κB inhibitors (BAY11‐7028) respectively and found that the effect of HRG on ROS levels in neutrophils could only be eliminated if both the MAPK and NF‐κB signalling pathways were inhibited (Figures 6F and G), and the regulatory effects of HRG on citH3 can be eliminated by simultaneously using anti‐IL‐8 antibody and BAY11‐7028 (Figure 6E). These results suggest that HRG affects NETs by combining MAPK and NF‐κB signalling pathways to regulate ROS levels in neutrophils. In addition, we tested the role of BAY11‐7028 in the context of neutrophil chemotaxis assay and found that BAY11‐7028 eliminated the effect of HRG on neutrophil chemotaxis (Figures S8a and b).

### 2.7 HRG enhances the efficacy of immunotherapy

Considering that HRG inhibits the production of NETs, we propose the hypothesis that HRG can improve the efficacy of ICB. We inoculated Hepa1‐6 cells into C57BL/6 mice to construct subcutaneous graft tumours. Remarkably, mice that HRG intraperitoneal injection exhibited a better response to the anti‐PD‐1 mAb as the impaired tumour growth and smaller tumour volumes (Figures 6H and I). This suggests that the combination of HRG and anti‐PD1 can enhance the efficacy of immunotherapy (7).

**FIGURE 7:**
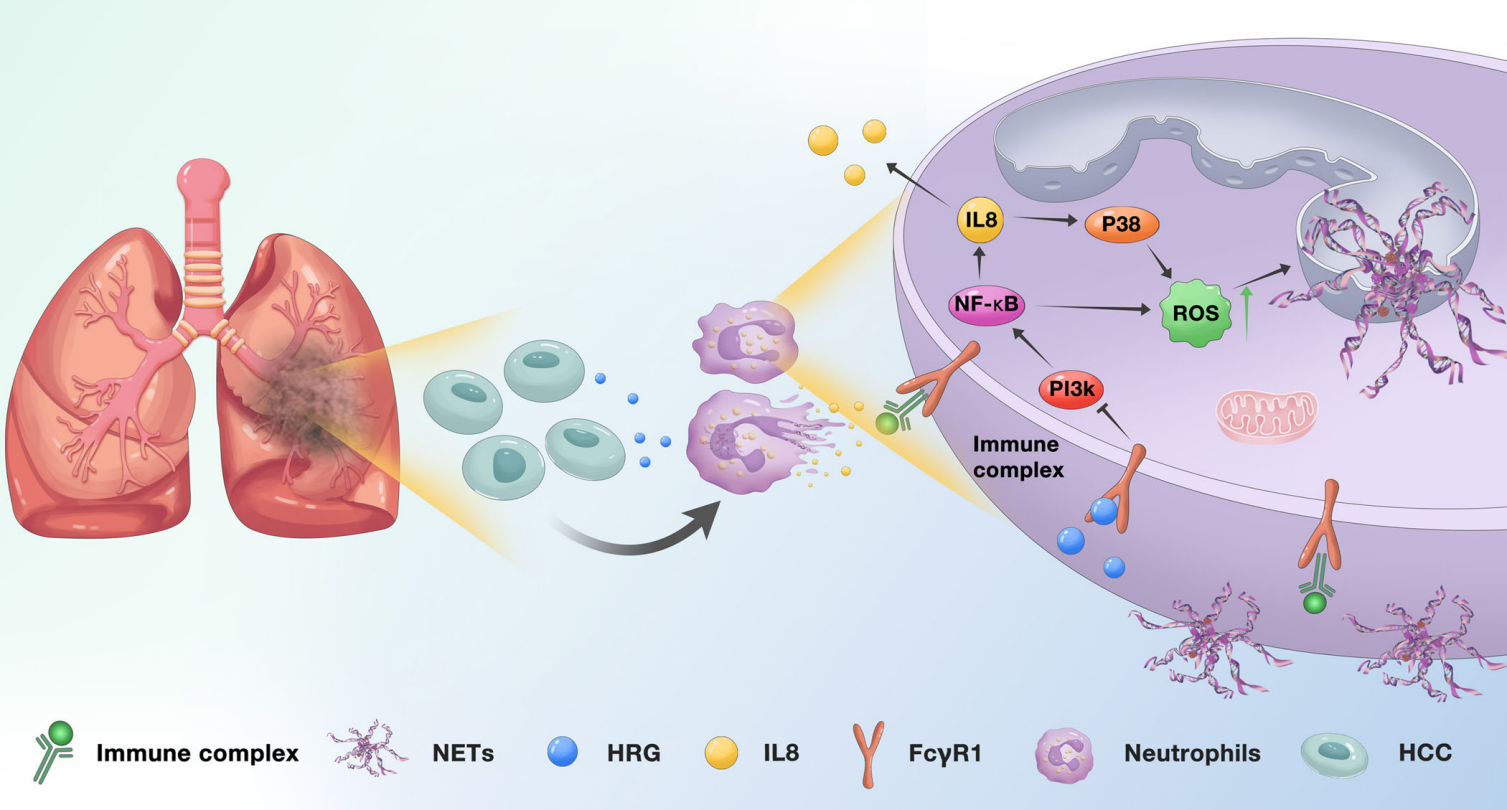
System illustration of HRG inhibiting liver cancer lung metastasis by modulating neutrophil and neutrophil extracellular trap formation.

## 3 DISCUSSION

In this study, we reported that HCC cells reduce the secretion of HRG, regulate the recruitment and activation of neutrophils in the metastatic microenvironment and promote the production of NETs, thereby promoting liver cancer lung metastasis. HRG binds to FCγR1 on the neutrophil membrane while inhibiting PI3K and NF‐κB activation, thereby reducing IL‐8 secretion to reduce neutrophil recruitment. Meanwhile, HRG inhibited IL‐8‐MAPK and NF‐κB pathway activation and ROS production, resulting in reduced NETs formation.

Secretory proteins produced by cancer cells are an important mediator for cancer cells to regulate the metastatic microenvironment.^31^, ^32^ We first identified HRG, a secreted protein associated with metastasis. By analysing a clinical cohort of patients, we found that HRG expression levels were significantly decreased in liver cancer tissues relative to normal liver tissues and that the risk of lung metastasis was higher in patients with low HRG expression levels. We observed that HRG is a key regulator of lung metastasis in HCC in vivo experiments but not in vitro experiments and that HRG is associated with diverse immune cells in the tumour microenvironment. These findings suggest that cancer cells alter the metastatic microenvironment by reducing HRG secretion and thus promote their own metastasis. In this regard, further studies are needed to address the causes of reduced HRG production in HCC cells. In addition, all animal experiments were performed in an intravenous setting and not in an orthotopic setting, so further in orthotopic experiments or other cancer species are needed to expand the scope of the conclusions.

The underlying mechanism of repressed expression of HRG in cancer cells may be partly attributed to the activation of NF‐κB signalling. A large number of studies have shown that the NF‐κB signalling can promote the development of tumours. Previous study revealed that NF‐κB binds to Farnesoid X receptor (FXR) and subsequently inhibits its transcriptional activity.^33^, ^34^ Additionally, HRG was proven a transcriptional target gene of FXR in liver cells.^35^ HRG expression might be suppressed by NF‐κB activation via the inhibition of FXR. The mechanism can partly explain that HRG down‐regulates in cancer cells. More potential mechanisms remain to be explored.

In the early stage of metastatic cancer, neutrophils are crucial for establishing a favourable microenvironment.^36^, ^37^ We observed that HRG inhibited neutrophils rather than other immune cells in the early lung metastatic microenvironment (vein injection 3 days). HRG suppressed neutrophil activity, thereby reducing neutrophil recruitment in the metastatic microenvironment. In the mechanism, FCγR1 is a receptor required for neutrophil maintenance activity,^38^ we found that HRG bound to FCγR1 in the neutrophil membrane and potently inhibited neutrophil and NETs formation in vitro. However, how HRG binds to FCγR1 in the neutrophil membrane and inhibits downstream pathways deserves further exploration. The regulatory effect of HRG on liver cancer lung metastasis was achieved by depleting Ly6G+ cells in a metastasis model relying on neutrophils in the pre‐metastatic microenvironment, this suggested an important role for neutrophils and NETs in early lung metastasis. However, HRG was also observed to be associated with M1 and NK cells in patient tumour tissues, so more research is needed to elucidate HRG effect on the microenvironment.

Previous research reveals that NETs regulate adaptive anti‐tumour immune evasion. NETs block contact between cytolytic cytotoxic T lymphocytes (CTLs), natural killer (NK) cells and surrounding tumour cells and reduce efficacy of immunotherapy. Mechanically, NETs wrap tumour cells to protect them from CTLs and NK cell‐mediated cytotoxicity.^24^ Considering that HRG inhibits the production of NETs, we propose the hypothesis that HRG can improve the efficacy of ICB. In vivo experimental results showed that the combination of HRG and anti‐PD1 can enhance the efficacy of immunotherapy. These results reveal that HRG has great clinical treatment value.

In conclusion, our study uncovered the mechanism by which liver cancer regulates neutrophil recruitment and NETs formation in the metastatic microenvironment by reducing HRG secretion, thereby promoting tumour lung metastasis. This suggests that HRG may have great potential in inhibiting tumour metastasis and recurrence. These data establish the value of HRG as a prognostic and therapeutic target. The results of this study will contribute to the development of possible strategies for treating metastases.

## 4 MATERIALS AND METHODS

### 4.1 Human tissues and blood samples

Formalin‐fixed paraffin‐embedded specimens from patients of liver cancer (*n* = 234) with following‐up information; another independent cohort of liver cancer (*n* = 80); primary and metastases specimens from the same liver cancer patients with lung metastases (*n* = 17) and peripheral blood of patients with HCC or HDs were obtained from Shanghai Renji hospital after informed consent by the patient. The approval was issued by the Shanghai Renji hospital's ethics committee, Shanghai Jiao Tong University. Clinical and pathological characteristics of patients with liver cancer were listed in the Table 1 (Supplementary methods).

### 4.2 Animal and animal models

HRG deficient mice on a C57BL/6 background were obtained from Willi Jahnen‐Dechent (University Hospital Aachen, Germany). Littermates of 8−10 weeks were used in experiments. The Shanghai Laboratory Animal Co., Ltd., China, provided C57BL/6 and athymic nude mice of 8−10 weeks. All experiments used mice of the same gender and age. Animal study approval was obtained from the Institutional Animal Care and Use Committee of Shanghai Jiao Tong University School of Medicine (Shanghai, China).

Mice were intravenously injected with 2 × 10^5^ cancer cells and samples of lung were harvested at 3 days and 5 weeks after injection. Intravenous injection of SK‐hep1 with HRG overexpression in athymic mice, HCC‐LM3 with HRG knockdown in athymic mice and Hepa 1−6 in C57BL/6 mice for lung metastasis analysis.

Neutrophils depletion was achieved by i.p. with 200 g/mice of Ly6G‐antibody or IgG control every 2 days before the injection of cancer cells until harvesting. The DNase 1 treatment involved i.p. with DNase 1 (5 mg/kg) 1 day before the injection of cancer cells until a week, and then twice a week. Bioluminescent imaging was acquired by IVIS Lumina II system.

### 4.3 Cell lines

SK‐hep1 and HCC‐LM3 were obtained from the Chinese Academy of Sciences. Hepa1‐6 was obtained from the Liver Cancer Institute, Fudan University. The cell lines generated in this study were grown in DMEM with 10% v/v FBS and 100 μg/ml penicillin/streptomycin. Cell cultures at passages 5−8 were used and tested for mycoplasma contamination weekly.

### 4.4 Isolation of neutrophils

Neutrophils were separated from the peripheral blood of donors using density gradient centrifugation with Histopaque 1077/1119. During centrifugation, peripheral blood was layered on top of Histopaque 1077/1119 and centrifuged at 600×*g* for 25 min at 24°C without braking. Analyses by flow cytometry and Trypan blue exclusion confirmed 90% purity and 95% viability. Neutrophils were cultured with RPMI 1640 medium containing 0.1% BSA unless otherwise noted.

### 4.5 Statistical analyses

GraphPad Prism 7.0 (GraphPad Software, La Jolla, USA) was used for data analysis. The difference between two groups was compared using Student's *t*‐test. For correlation analysis, Pearson correlation test was used. For follow‐up data, log‐rank tests were used and *p* values < .05 were considered as statistically significant.

Materials and method details are provided in Supporting Information.

## CONFLICT OF INTEREST STATEMENT

There are no conflicts of interest to declare.

## Supporting information

Supporting Information

Supporting Information
