# Supplementary material for: HRG inhibits liver cancer lung metastasis by suppressing neutrophil extracellular trap formation
Source: Clin Transl Med. 2023 May 30;13(6):e1283. doi: 10.1002/ctm2.1283 (PMC10230156; doi:10.1002/ctm2.1283)
Supplement: Supplementary file 1 — Supporting Information [file CTM2-13-e1283-s002.docx]

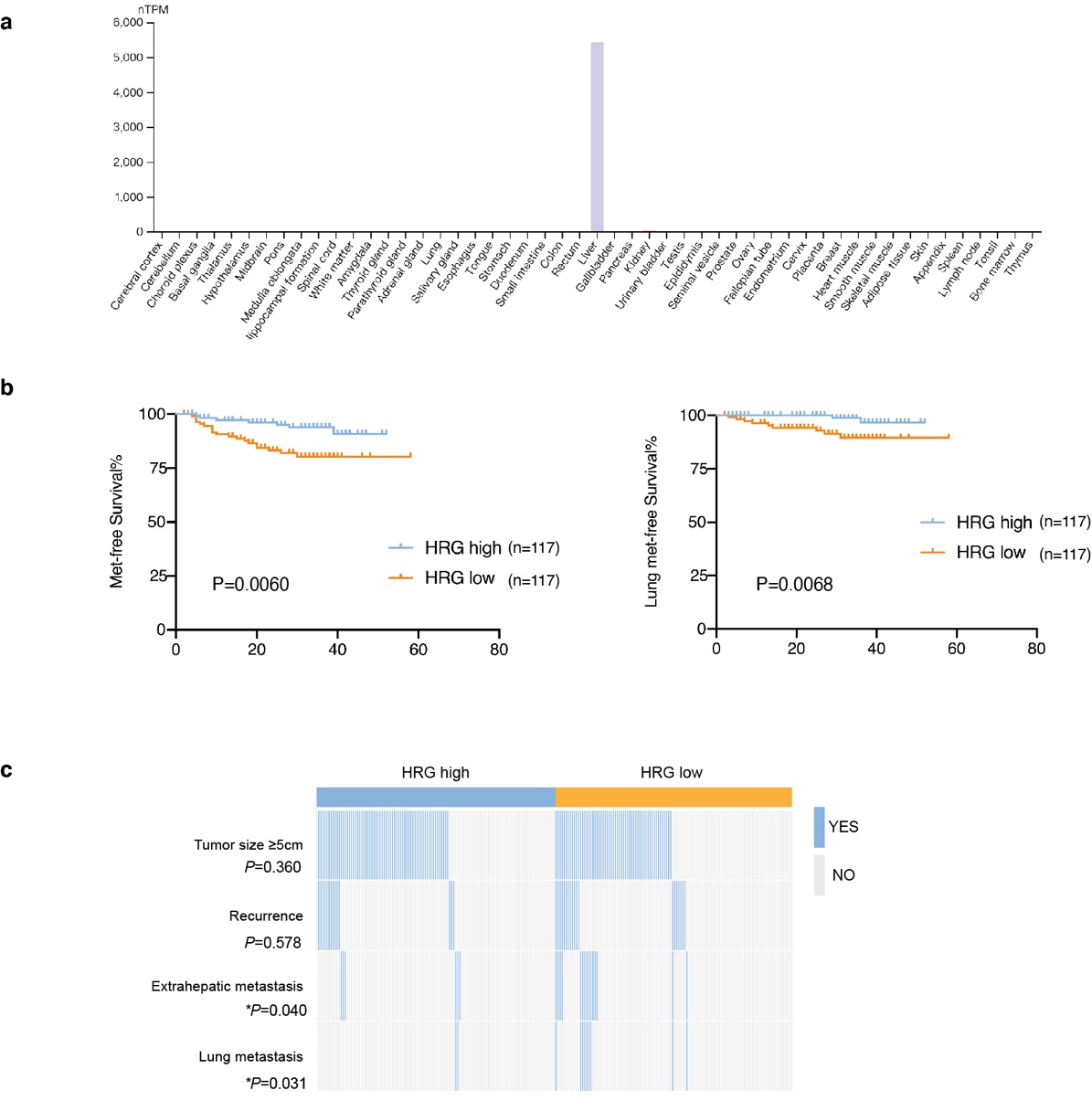


**Supplementary Fig. 1 HRG expression is associated with liver cancer lung metastasis immune microenvironment**

(a) Expression of HRG in different organs based on HPA RNA-seq database.

(b-c) HRG expression analysis of a liver cancer cohort from Shanghai Renji Hospital (n=234). (b) clinical events of HRG high group (n=117) and HRG low group (n=117) and (c) Metastasis-free survival and lung metastasis-free survival analysis of a liver cancer cohort from Shanghai Renji Hospital based on HRG expression level.


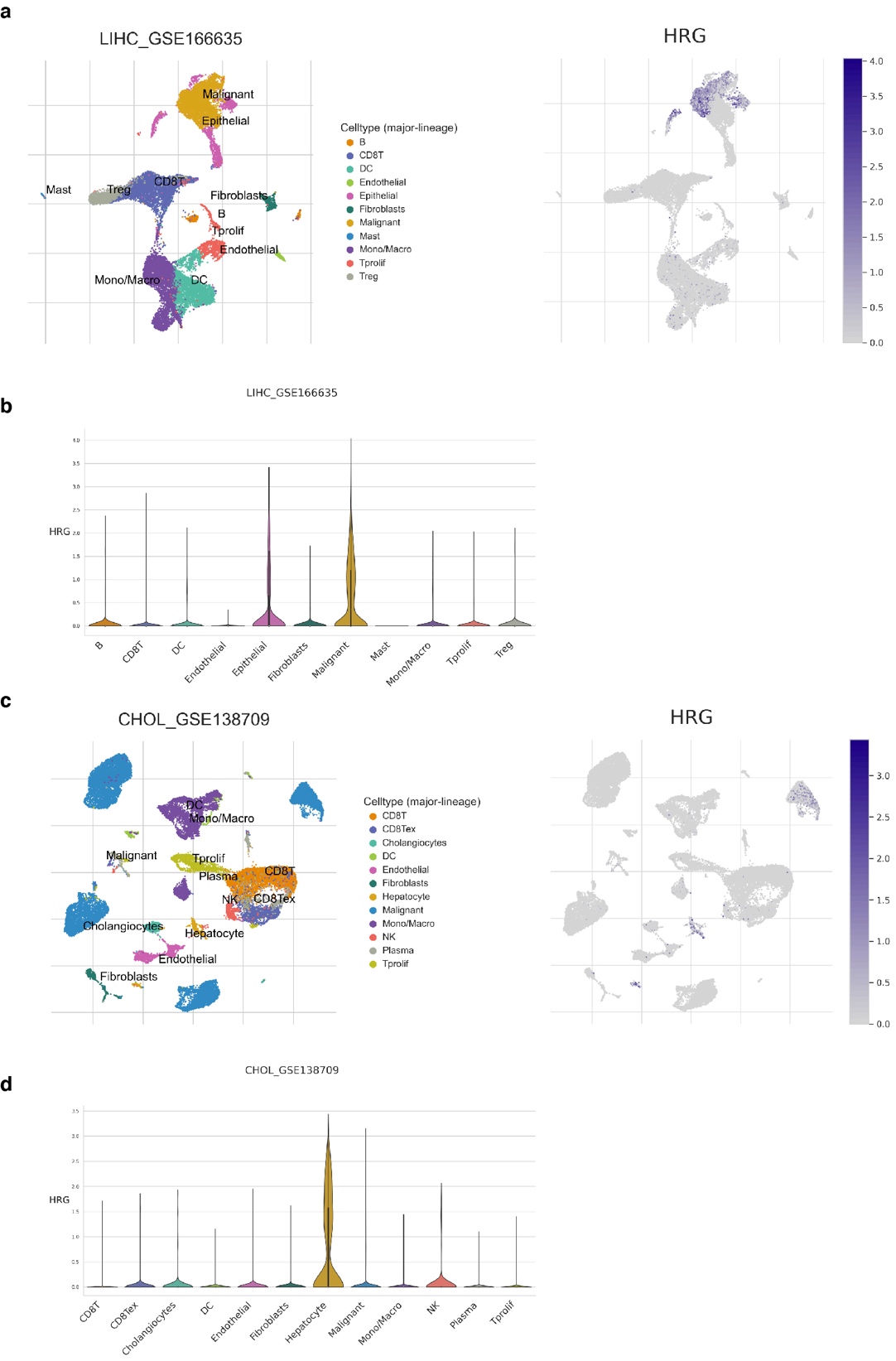


**Supplementary Fig. 2 The distribution of HRG in HCC and CCA using single cell RNA-seq database**

(a, b) The distribution of HRG expression in different cell types in GSE166635. (a) Umap. (b) Violin plot.

(c, d) The distribution of HRG expression in different cell types in GSE138709. (a) Umap. (b) Violin plot.


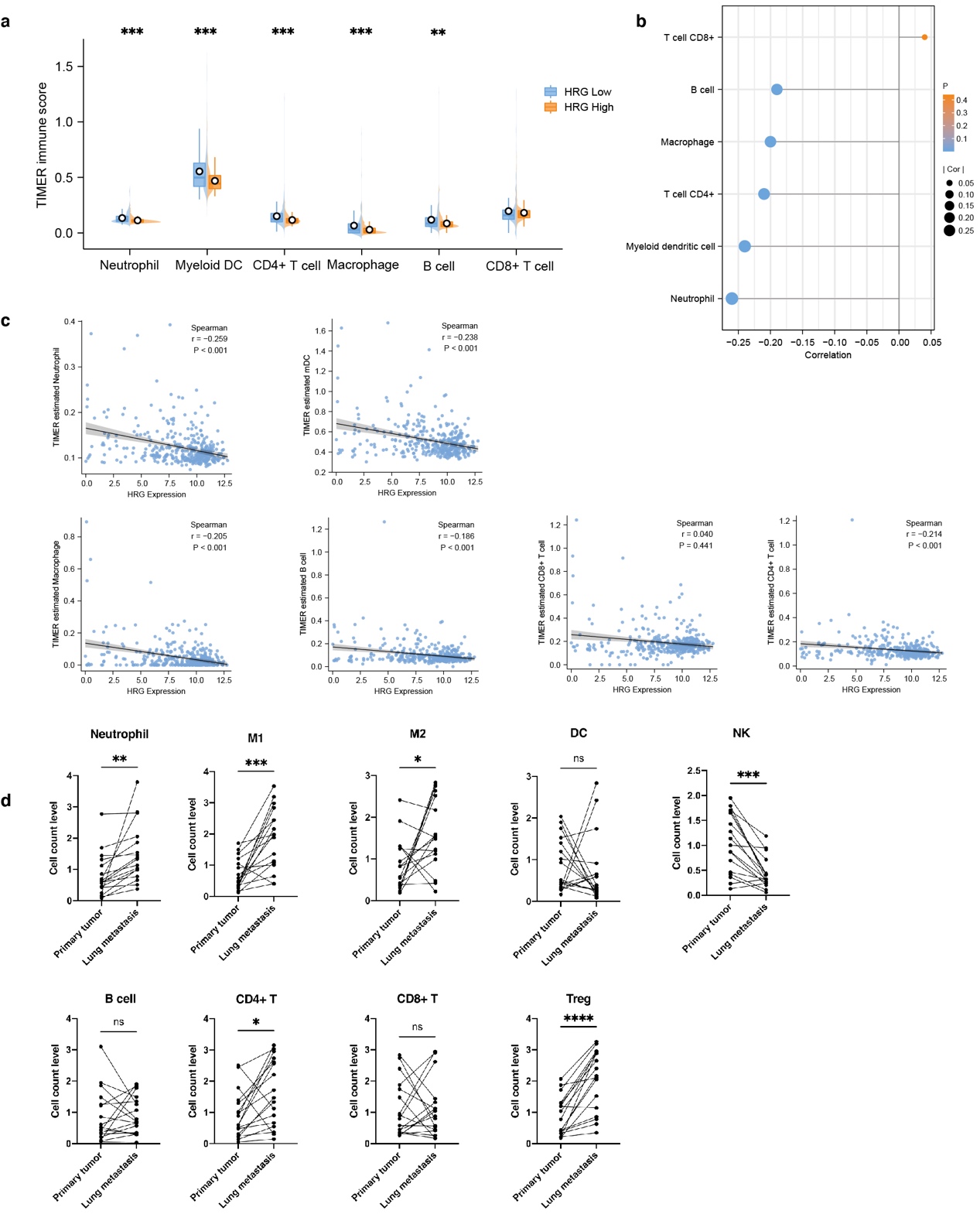


**Supplementary Fig. 3 HRG is associated with tumor immune microenvironment**

(a, b) Analyses of HRG expression and immune infiltration in LIHC of The Cancer Genome Atlas (TCGA). (a) TIMER immune score of infiltration profile of immune subsets based on HRG expression levels from TCGA. (b) Correlation between HRG expression and immune infiltration in LIHC of TCGA dataset based on the TIMER immune score.

(c) Correlation analysis of HRG and immune cells from TCGA database based on TIMER.

(d) Altered immune cells in paired primary tumors and lung metastases form the same patients (n=17).


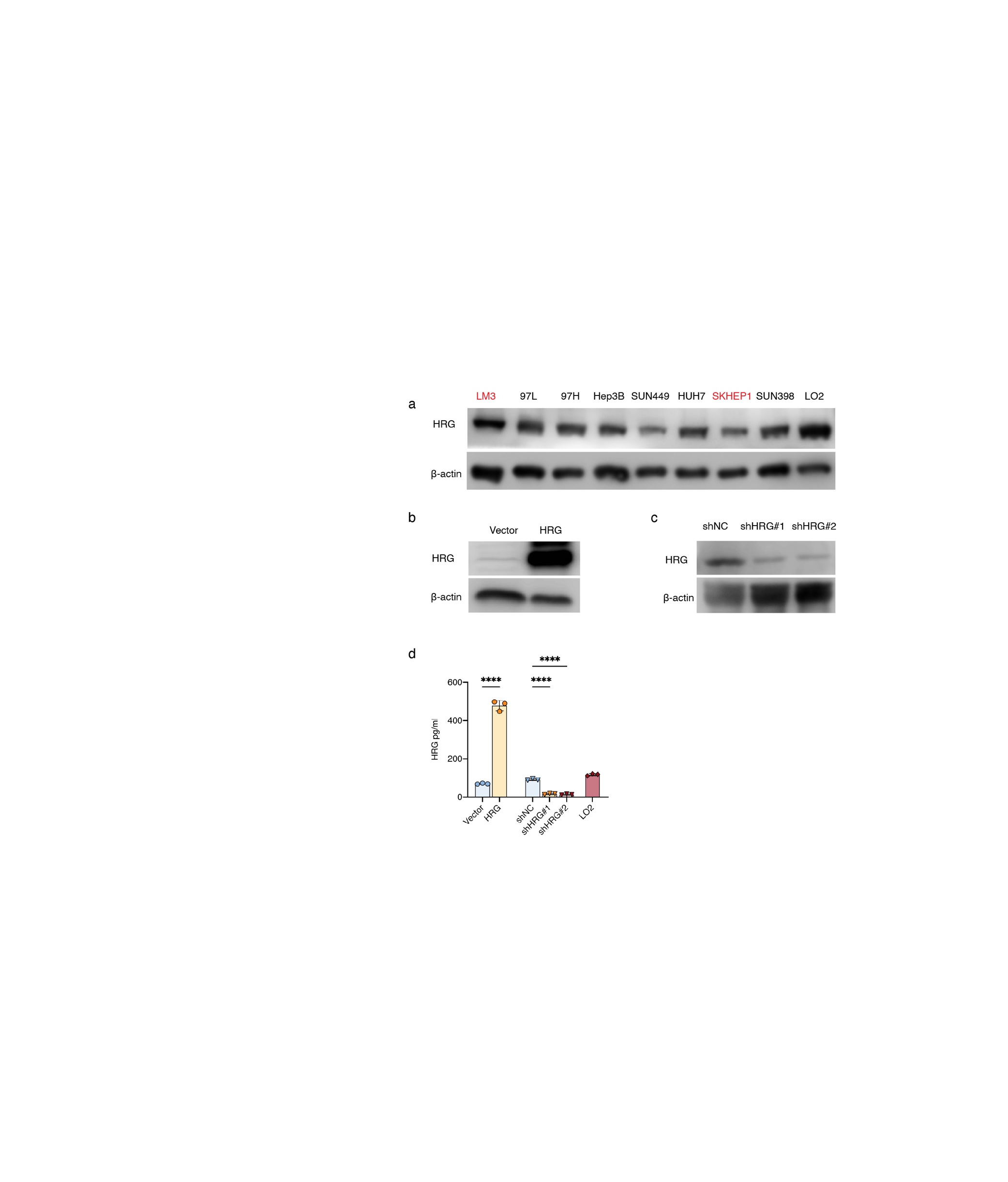


**Supplementary Fig. 4 Construction of HRG overexpressed and knockdown cell lines**

(a) HRG expression level in HCC cell lines. HRG expression was lower in SKHEP1 and higher in LM3. (b) HRG overexpression in SKHEP1 cell line. (c) HRG knockdown in LM3 cell line.

(d) HRG in conditioned-medium of HCC cells and normal liver cells was detected by ELISA.


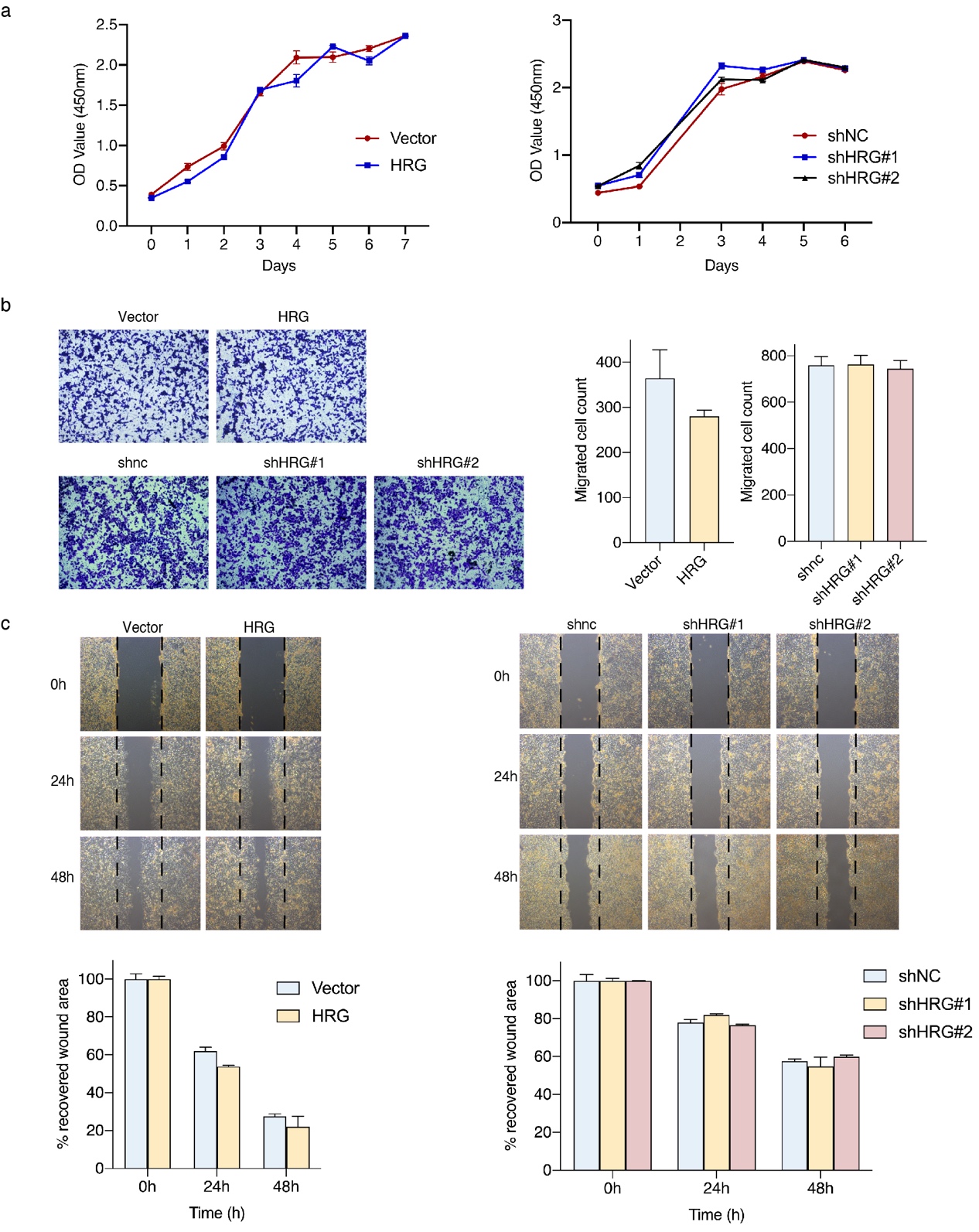


**Supplementary Fig. 5 HRG has no effect on cancer cell in vitro**

(a-c) In vitro analysis of cancer cell (a) CCK-8 experiment for proliferation, (b) transwell experiment and (c) wound-healing migration.


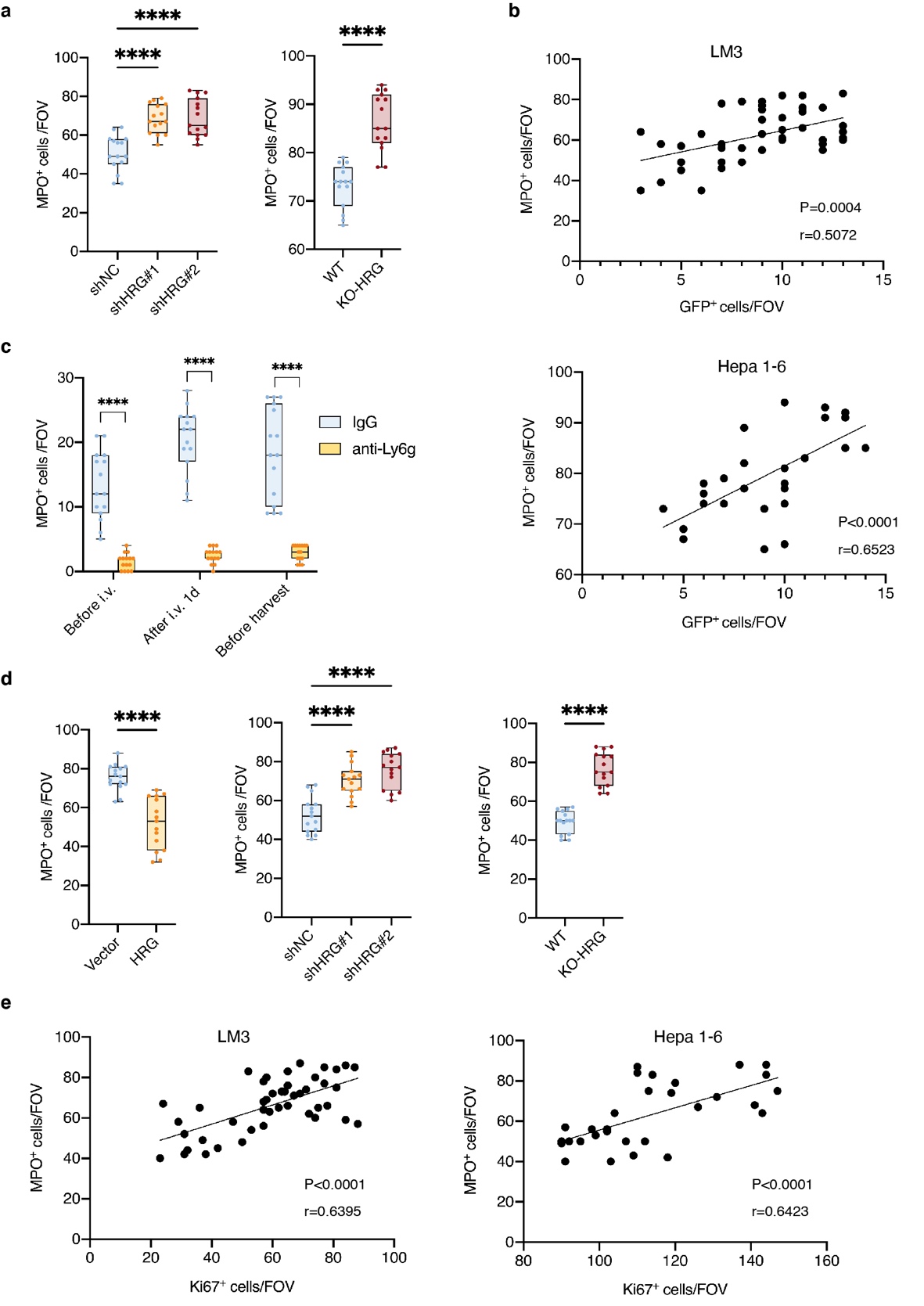


**Supplementary Fig. 6 HRG decreases neutrophil in lung metastasis niches**

(a, b) Analyses of alternations of immune cells in early lung metastases at 72h after intravenous injection by (a) IF analyses of MPO^+^ neutrophils and (b) correlation of infiltrated MPO^+^ neutrophils with GFP^+^ cancer cells (n=15 RMFs from 3 mice per group).

(c) The neutrophil depletion efficacy before tumor inoculation, after tumor inoculation 1 day and before harvesting samples. IF analyses of MPO^+^ neutrophils.

(d, e) Analyses of alternations of immune cells in lung metastases at 5w after intravenous injection

by (d) IF analyses of MPO^+^ neutrophils and (e) correlation of infiltrated MPO^+^ neutrophils with Ki67^+^ cells (n=15 RMFs from 3 mice per group).


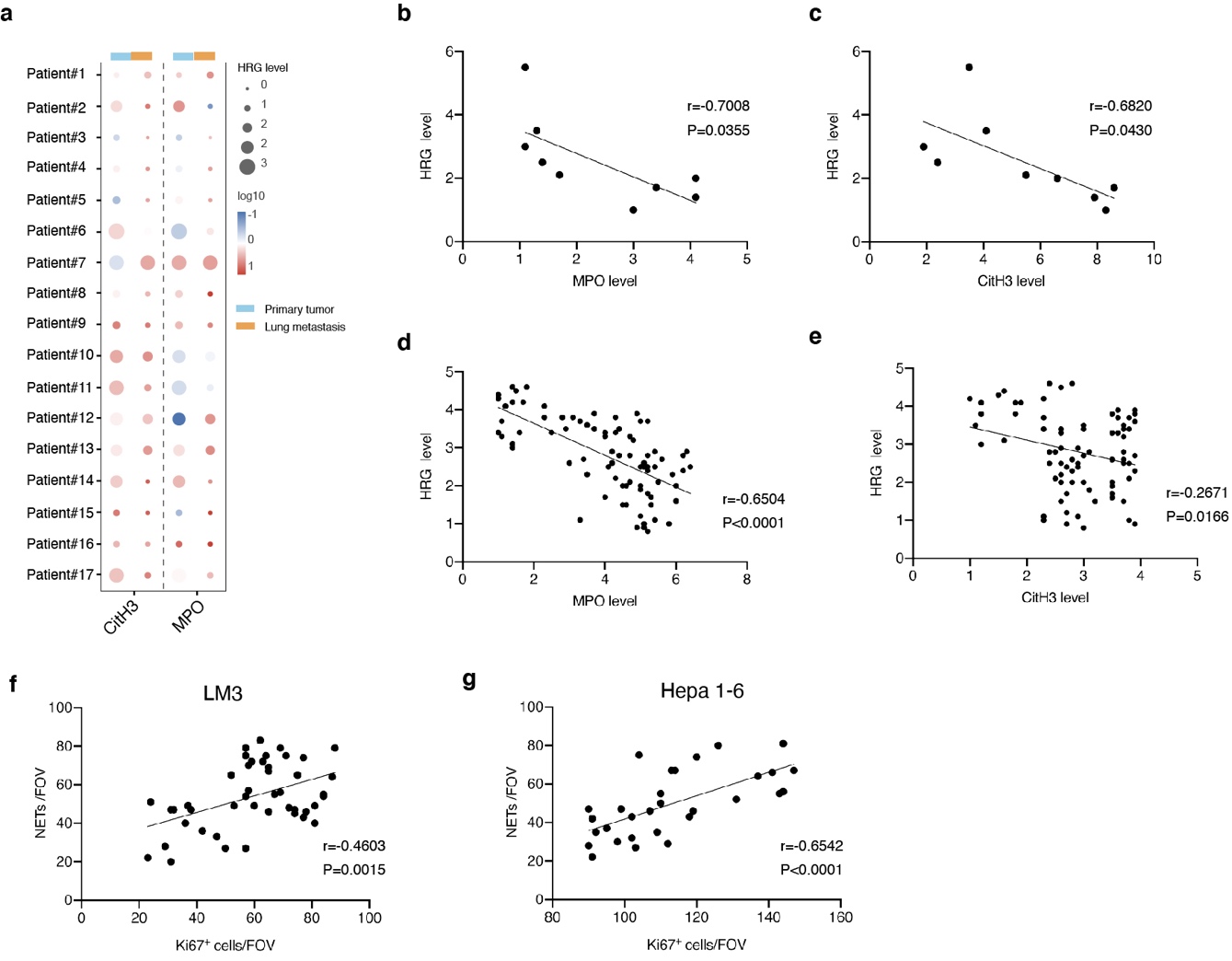


**Supplementary Fig. 7 HRG regulates liver cancer lung metastasis by inhibiting neutrophils to form NETs**

(a) Dot plots displaying HRG expression level, MPO^+^ neutrophils and NETs in paired primary tumors and lung metastases.

(b, c) Correlation analysis of (b) HRG level with MPO level and (c) HRG level with CitH3 level in 9 extrahepatic metastases (6 bone metastases and 3 abdominal metastases).

(d, e) Correlation analysis of (d) HRG level with MPO level and (e) HRG level with CitH3 level in independent cohort of patients with liver cancer (n=80).

(f, g) Correlation analysis of NETs formation and proliferative cancer cells (Ki67+ cells) in LM3 and Hepa1-6 cell lines.


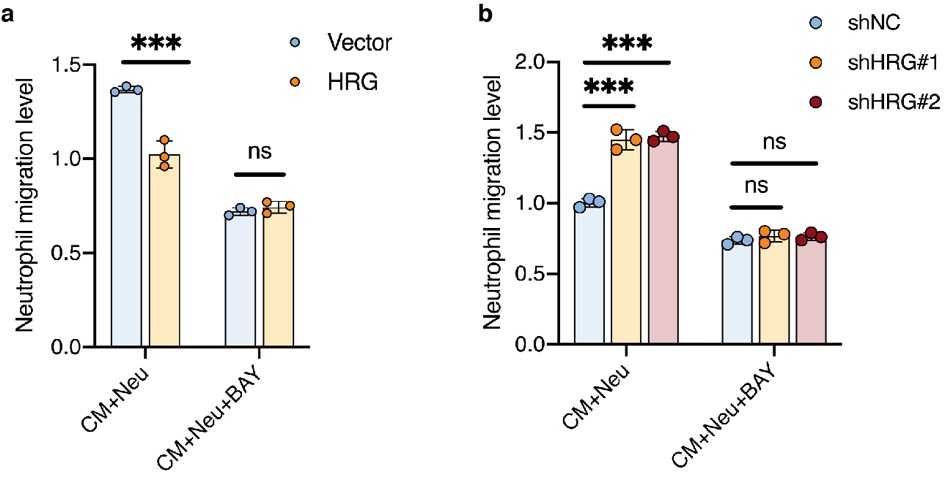


**Supplementary Fig. 8 BAY11-7028 eliminated the effect of HRG on neutrophil chemotaxis**

(a, b) Migration of human neutrophils recruited by conditioned medium (CM) of SK-hep1 (HRG overexpression) or LM3 (HRG knockdown) and medium from neutrophils (Neu) pre-treated with CM for 8 hours with or without BAY11-7028 (n=3).
