# Supplementary material for: HRG inhibits liver cancer lung metastasis by suppressing neutrophil extracellular trap formation
Source: Clin Transl Med. 2023 May 30;13(6):e1283. doi: 10.1002/ctm2.1283 (PMC10230156; doi:10.1002/ctm2.1283)
Supplement: Supplementary file 2 — Supporting Information [file CTM2-13-e1283-s001.docx]

Online data acquisition and analyses

We performed differential gene analysis for 10 primary liver cancer, 19 extrahepatic metastasis, 13 lung metastases using the Gene Expression Omnibus (GEO) dataset (GSE40367). We obtained RNA sequencing data from 3 primary cell lines (PLC/PRF/5, JHH1, HuH7) and 2 metastasis cell lines (SK-HEP-1, JHH2) from the Cancer Cell Line Encyclopedia (CCLE). SK-HEP-1 is derived from the metastatic site (ascites fluids) of a patient with adenocarcinoma of the liver. JHH2 is derived from metastatic site (ascites fluids) of a patient with hepatocellular carcinoma. Data analysis was done under package limma Fold change > 1 and adj. p < 0.05 were regarded as the cut-offs for DEGs. All the statistics were operated and extracted with R version 3.6.2 (R Foundation for Statistical Computing, Vienna, Austria) and Bioconductor. The distribution of HRG in HCC (GSE166635) and CCA (GSE138709) using single cell RNA-seq database (http://tisch.comp-genomics.org).

Western blot

Cells were collected by scraper or trypsinized and then lysed with lysis buffer (50 mM Tris-HCl, 150 mM NaCl, 1% NP-40 detergent, 0.5% sodium deoxycholate, 0.1% SDS with phosphatase and protease inhibitors), and homogenized in RIPA buffer on ice for 30 min, followed by centrifugation at 14,000 g for 10 min. The protein concentration was measured with a BCA Protein Assay Kit (Thermo Fisher Scientific). Protein samples were separated on 10% SDS-polyacrylamide gel and transferred onto 0.22 μm NC membranes. The membranes were washed, blocked, and incubated with primary antibodies to HRG (Invitrogen, 1:1000), FcγR1 (Abcam, 1:1000), p-PI3K (Abcam, 1:1000), PI3K (Abcam, 1:1000), CitH3 (Abcam, 1:1000), p65 (CST, 1:1000), p-p65 (CST, 1:1000), p-p38 (CST, 1:1000), p38 (CST, 1:1000), src (CST, 1:1000), p-src (CST, 1:1000) and β-actin (Sigma, 1:10000) in 4 ℃ overnight, then the blots were washed, incubated with secondary antibodies, and detected by ECL assays.

Flow cytometry

Briefly, lung metastases were picked, minced, further digested by 5 mg/ml Collagenase Type II (Sigma) and 0.001% (W/V) DNase I (Sigma) at 37 °C for 30min to prepare single cell suspensions. Then, red blood cells were lysed with RBC lysis reagent (Sigma). Single cell suspensions were incubated for 30 min at 4 °C with BV510 anti-mouse CD45 (BD, 1:100), APC-Cy7 live/dead (BD, 1:100), BV786 anti-mouse CD11b (BD, 1:100), BV421 anti-mouse F4/80 (BD, 1:100), FITC anti-mouse CD86 (BD, 1:100), PE anti-mouse CD206 (BD, 1:100), PE-Cy7 anti-mouse Ly6G (BD, 1:100), PerCP-Cy5.5 anti-mouse Ly6C (BD, 1:100), BV650 anti-mouse CD11c (BD, 1:100), APC anti-mouse NK1.1 (BD, 1:100), APC anti-mouse CD45 (Biolegend, 1:100), FITC anti-mouse CD11b (Biolegend, 1:100), PE anti-mouse Ly6G (Biolegend, 1:100), FITC anti-mouse CD45 (Biolegend, 1:100), PE anti-mouse F4/80 (Biolegend, 1:100), PC7 anti-mouse CD206 (Biolegend, 1:100), APC anti-mouse CD86 (Biolegend, 1:100), PC7 anti-mouse CD11c (Biolegend, 1:100), and PC5.5 anti-mouse CD3 (Biolegend, 1:100). Flow cytometry was performed in BD Fortessa FACS with Diva software v6.0. and quantified by the FlowJo V10 software.

In vitro NETs analysis

For NETs analysis, neutrophils (2.5 × 10^5^cells) were seeded on 24-well plates coated with poly-L-lysine (Sigma) for 4h after adding PMA (Sigma, 20nM) or 10% cancer cell CM. Cell-impermeable DNA dye SYTOX Green (Thermo Fisher Scientific, 1μM) and cell-permeable DNA dye SYTO Red (Thermo Fisher Scientific, 1μM) were added to the incubation system. At the end of incubation, the plates were directly moved to the fluorescence microscope (Leica) for NET formation visualization. NETs were evaluated as the percentage covered by a positive staining area.

Isolation of neutrophil plasma membrane proteins

Plasma membrane proteins were isolated from human peripheral blood derived neutrophils using the Pierce Cell Surface Protein Isolation Kit (Thermo Scientific) according to the manufacturer's protocols. Briefly, the cells were labeled with Thermo Scientific EZ-Link Sulfo-NHS-SS-Biotin. Then, the whole cell lysate was obtained by lysing with Pierce IP Lysis Buffer and sonicating on ice using five 1-second bursts. The biotinylated cell membrane proteins were purified by NeutrAvidin-agarose resin and released by incubation with Pierce IP Lysis Buffer containing 5 mM dithiothreitol.

Nano-HPLC-MS/MS analysis

The peptides were re-dissolved in 0.1% formic acid in water and analyzed by Orbitrap Fusion coupled to an EASY-nanoLC 1200 system (Thermo Fisher Scientific, MA, USA).

Cytokine analysis

Cytokines were detected in media of neutrophils cultured with CM from neutrophils of patients with HCC by the luminex liquid suspension chip assay, which was performed by Wayen Biotechnologies (Shanghai, China) with Bio-Plex MAGPIX System (Bio-Rad) in accordance with the manufacturer’s instructions.

Cytokines were detected in media of neutrophils cultured with CM from neutrophils of healthy donors using the human Cytokine Antibody Array (ab133997, Abcam) according to the manufacturer’s instruction. Briefly, membranes were blocked with the blocking buffer for 45 min, then incubated overnight at 4 °C with 1 ml of samples containing the protease inhibitor cocktail. After biotin-conjugated antibody and HRP-streptavidin incubation, chemiluminescence detection was performed.

Immunofluorescence (IF) staining

For murine lung tissues, the lungs were perfused with 50 ml of PBS through the right ventricle until they were cleared of blood. Then, the tissues were rinsed with pre-chilled PBS and fixed in 4% PFA for 2h at 4 °C on a shaker, dehydrated in 30% sucrose in PBS overnight, embedded in OCT (Sakura) for 1h at 4 °C, followed by freezing at -80 °C. Tissues were sectioned to 10 μm thickness, washed twice with PBS, permeabilized in 0.1% Triton X-100 for 15 min, and blocked in PBS containing 5% BSA for 45 min.

MPO and CitH3 levels was determined by immunofluorescence staining and the number of MPO+ and CitH3+ cells was counted under high power field.

Multiplex immunohistochemistry (mIHC) staining

We performed the fluorescent dyes by using the CD11c, CD86, CD206, CD66b, CD14, CD11b, CD19, CD8, CD4, Foxp3, CD56 anti-human antibody (Abcam). We scanned the slides using the PerkinElmer Vectra3® platform and quantified the results by using PerkinElmer Vectra3® platform.

ROS analysis

To measure the ROS levels, human neutrophils were cultured in cancer cell CM or non-conditioned medium, and treated with DMSO or various inhibitors, including BAY11-7082 (10 μM) and SB203580 (10 μM) for 1 h at 37 °C. Cells were then harvested and resuspended in assay buffer containing 10 μM CM-H2DCFDA (Invitrogen), incubated for 20 min at 37 °C in the dark, washed with pre-chilled PBS, followed by FACS analysis within 30 min.

Further details of materials and methods are described in Additional files

**Table 1: Clinicopathological features of 314 patients with HCC**

| Characteristic | Number of cases | Percentage |
| --- | --- | --- |
| Gender |  |  |
| Male | 153 | 48.7 |
| Female | 161 | 51.3 |
| Age |  |  |
| ≤60 | 86 | 27.4 |
| ＞60 | 228 | 72.6 |
| Size (mm) |  |  |
| ＜50 | 221 | 70.4 |
| ≥50 | 93 | 29.6 |
| Vascular involvement |  |  |
| Yes | 61 | 19.4 |
| No | 253 | 80.6 |
| AJCC Stage |  |  |
| 1 | 64 | 20.4 |
| 2 | 132 | 42.0 |
| 3-4 | 118 | 37.6 |
| Cirrhosis |  |  |
| Positive | 142 | 45.2 |
| Negative | 172 | 54.8 |
| HBsAg |  |  |
| Positive | 242 | 77.1 |
| Negative | 72 | 22.9 |
| Neutrophil |  |  |
| Cold | 33 | 10.5 |
| Non-Cold | 281 | 89.5 |
